# Adaptations for centromere function in meiosis

**DOI:** 10.1042/EBC20190076

**Published:** 2020-05-14

**Authors:** Reinier F. Prosée, Joanna M. Wenda, Florian A. Steiner

**Affiliations:** Department of Molecular Biology and Institute for Genetics and Genomics in Geneva, Section of Biology, Faculty of Sciences, University of Geneva, 1211 Geneva, Switzerland

**Keywords:** CENP-A, centromere, chromosomes, meiosis

## Abstract

The aim of mitosis is to segregate duplicated chromosomes equally into daughter cells during cell division. Meiosis serves a similar purpose, but additionally separates homologous chromosomes to produce haploid gametes for sexual reproduction. Both mitosis and meiosis rely on centromeres for the segregation of chromosomes. Centromeres are the specialized regions of the chromosomes that are attached to microtubules during their segregation. In this review, we describe the adaptations and layers of regulation that are required for centromere function during meiosis, and their role in meiosis-specific processes such as homolog-pairing and recombination. Since female meiotic divisions are asymmetric, meiotic centromeres are hypothesized to evolve quickly in order to favor their own transmission to the offspring, resulting in the rapid evolution of many centromeric proteins. We discuss this observation using the example of the histone variant CENP-A, which marks the centromere and is essential for centromere function. Changes in both the size and the sequence of the CENP-A N-terminal tail have led to additional functions of the protein, which are likely related to its roles during meiosis. We highlight the importance of CENP-A in the inheritance of centromere identity, which is dependent on the stabilization, recycling, or re-establishment of CENP-A-containing chromatin during meiosis.

## Introduction

In dividing cells, the genome duplicated during DNA replication has to be equally and faithfully segregated to the daughter cells. More than a century ago, Walther Flemming used salamander embryos to carefully observe the dynamics of chromosomes during cell division [1]. He described a defined region within each chromosome that was especially dense—a chromatin feature we now refer to as the centromere. Centromeres form sites called primary constrictions, where sister chromatids are held together after condensation in preparation for their segregation. The centromere forms the basis for the kinetochore, a proteinaceous structure that attracts microtubules and ensures the timely and faithful segregation of the sister chromatids during cell division.

About 140 years after their initial description, centromeres still hold many secrets. Despite being essential for cell division, centromeres have evolved different forms of organization. The three main types have been categorized: point centromeres (e.g. budding yeast), regional centromeres (e.g. humans), and holocentromeres (e.g. roundworms) [2,3]. These forms of centromeres vary in terms of their spatial organization, occupying either a single point, a region, or the entire chromosome, respectively. This difference in organization is accompanied by remarkable meiotic adaptations, especially in the case of holocentricity [4]. With the exception of budding yeast point centromeres, DNA sequence was shown to be dispensable for centromere identity [5]. Instead, in most species centromeres are defined by a histone variant, called CENP-A, that acts as an epigenetic mark to establish and maintain functional centromeres [6].

The loading, maintenance, and function of CENP-A in centromeric nucleosomes have been intensively studied in mitotic cells, mainly using *in vitro* or cell culture model systems, and depend on an intricate network of protein factors and post-translational modifications [7,8]. In contrast, how centromeres function during meiosis, and how meiotic centromeres differ from mitotic centromeres is much less clear, as these questions need to be addressed in the context of whole organisms or with the use of isolated germ cells that are difficult to maintain in culture. Nevertheless, an increasing number of studies are shedding light on the dynamics of centromeres during meiosis, and help uncover the mechanisms by which CENP-A maintains its centromeric location despite the prolonged cell cycle arrest in female meiosis I. In this review, we aim to highlight studies that shape our understanding of the role of centromeres in meiosis.

## Roles of centromeres in meiosis-specific processes

**Figure 1:**
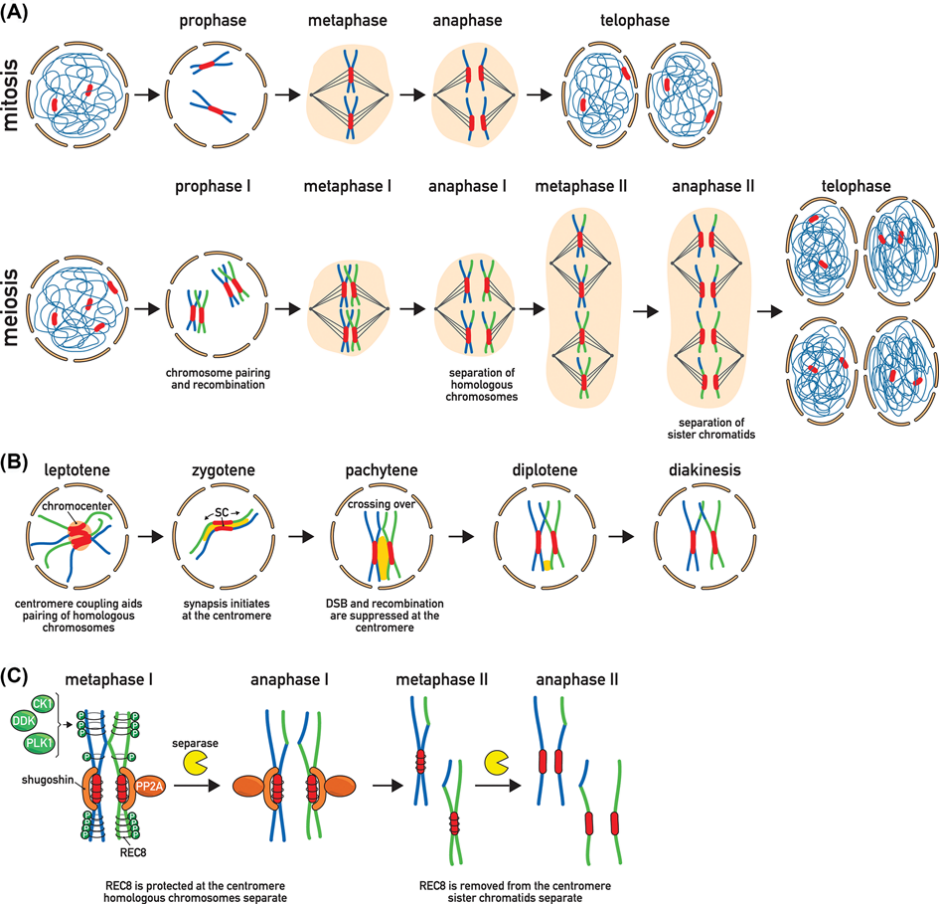
Meiosis-specific adaptations of centromeres (**A**) Overview of meiotic stages, highlighting the roles of centromeres (red squares) in chromosome pairing and recombination, and in segregation of homologous chromosomes and sister chromatids. (**B**) Overview of the stages of meiotic prophase I, detailing the roles of the centromere in chromosome pairing and recombination; SC, synaptonemal complex (in yellow). (**C**) Layers of regulation ensuring stepwise release of cohesin (REC8) in anaphase I and anaphase II. Phosphate groups are depicted as green circles with the label ‘P’; PLK1, polo-like kinase 1; CK1, Casein kinase 1; DDK, Dbf4-dependent kinase; PP2A, Protein phosphatase 2A.

For mitotically dividing cells, the ultimate goal is to ensure the equal and faithful segregation of its DNA, so the resulting daughter cells receive identical genomic information (Figure 1A). In meiosis, cell divisions have additional goals. Here, the cell also aims to recombine its DNA and reduce the genomic content by half (“reductional divisions”) in order to create haploid gametes (oocytes or sperm) for sexual reproduction. These goals are achieved by two subsequent divisions: the first results in the separation of homologous chromosomes and the second division separates the sister chromatids. Segregation of chromosomes during both divisions requires functional centromeres in most organisms (Figure 1A).

Only a few key processes distinguish meiosis from mitosis, which include homologous chromosome pairing, synapsis, and recombination, as well as the stepwise separation of the chromosomes. This is nicely illustrated by the *Arabidopsis thaliana MiMe (Mitosis instead of Meiosis)* phenotype in which three mutations (in *Atspo11-1, Atrec-8* and *osd-1*) are sufficient to disrupt these steps and revert meiosis back to mitosis [9]. Mutations of *Atspo-11* impair pairing, synapsis, and recombination of homologous chromosomes; *Atrec-8* mutations change the first meiotic division into a mitotic division; and *osd-1* mutants skip the second meiotic division. These meiosis-specific events have likely led to the emergence of roles of the centromere that go beyond chromosome separation and affect processes such as chromosome pairing, synapsis, and cohesion of homologs to allow the timely release of sister chromatids.

To allow recombination in early prophase I, chromosomes first need to move into close proximity of each other. During this process, centromeres act as preferential interaction sites for chromosomes (Figure 1B) [10]. This is known as centromere coupling, which was first described in budding yeast and involves the association between centromeres that can reside on either homologous or nonhomologous chromosomes [11]. Centromere coupling normally precedes centromere pairing, which refers only to associations between homologous chromosomes. The centromere is the main site for homologous chromosome pairing, although this process can also be achieved through the subtelomeric region, as shown in the common bread wheat *Triticum aestivum* [12]. While both centromere coupling and centromere pairing are important for recombination in later meiosis, they seem to rely on different molecular mechanisms, and are not dependent on each other [13,14]. In fact, centromere pairing is stable in mutants that affect centromere coupling [14].

Once centromeres or telomeres are paired, the whole chromosomes pair in a process known as synapsis. Centromeres have been shown to be primary initiation sites of synapsis in several species (Figure 1B) [15–18]. A study in budding yeast showed that the main component of the synaptonemal complex, Zip1, associates with centromeres. The polymerization of Zip1 at the centromere then starts synapsis, which spreads along the chromosome axes [15]. Synapsis can also occur at non-centromeric locations in budding yeast, but it is then mostly dependent on Zip3, a component of the synapsis initiation complex [15]. In *Drosophila melanogaster* oocytes, the C[3]G protein (a homolog of Zip1) forms foci that co-localize with clustered centromeres (called chromocenters). Like in yeast, the *D. melanogaster* chromocenter is not the only site of synapsis initiation [16,17]. Interestingly, the recombination events that follow synapsis during pachytene are suppressed close to the centromere [19,20]. In budding yeast, this repressive mechanism depends on multiple meiotic factors, including the ATPase Pch2, the origin recognition complex subunit Orc1, and the Ctf19 kinetochore complex [21,22]. These factors work at multiple levels to inhibit crossovers near the centromere, by preventing both meiotic double stranded breaks (DSBs) as well as inter-homolog repair [21,22]. In fission yeast *Schizosaccharomyces pombe*, heterochromatin plays a major role in the repression of meiotic recombination at the centromere. Strains deficient in the RNA interference pathway or histone H3K9 methyltransferase, which are both required for the maintenance of pericentric heterochromatin in *S. pombe*, showed a dramatic increase in DSBs formation and recombination at pericentric locations [23].

After recombination, the successful step-wise segregation of meiotic chromosomes, homologs during the first division and sister chromatids during the second division, relies on a functional meiotic spindle and the carefully controlled release of cohesion at the centromere (Figure 1C) [24,25]. In fission yeast, both telomeres and centromeres can promote the assembly of the meiotic spindle [26]. Clustered telomeres (telomere bouquet) stimulate the accumulation of Sad1, a crucial component for spindle formation, at the spindle pole body (the yeast equivalent of the centrosome). This ability is shared by the centromere, which enables both chromosomal structures to stimulate meiotic spindle formation and control proper centrosome behavior [26].

The controlled release of cohesion at the centromere is mostly dependent on the meiosis-specific cohesin component REC8 [27–29]. It has been implicated specifically in centromeric cohesion between sister chromatids, preventing their premature separation during the first meiotic division [28,30]. REC8 is present at centromeric sites until anaphase of meiosis II, at which time separase cleaves it and thereby enables separation of the sister chromatids (Figure 1C). There are intricate layers of control that shield REC8 from early removal, which rely heavily on phosphorylation events. Several protein kinases, including PLK-1, CK1 delta/epsilon, and DDK, as well as protein phosphatases (e.g. PP2A) regulate REC8 functioning at centromere [27,31–33]. These phosphorylation events are themselves controlled by additional protein factors, including Shugoshin, which is able to bind PP2A and counteract phosphorylation of centromeric REC8 [34,35].

The multi-layered regulation of REC8 is a good example of how centromeres have adapted to the specific needs of meiosis, while retaining their crucial role in chromosome segregation. Moreover, as illustrated by centromere coupling and pairing, the centromere has gained additional roles that are specific to prophase I of meiosis, aiding the successful recombination of homologous chromosomes.

## Meiotic drive

The centromere has not only acquired novel roles to aid meiosis, but also evolved features that have been likened to “selfish elements”. In many species, female and male meiosis differ in the amount of germ cells produced per meiotic cycle. In males, meiosis is symmetrical, resulting in four sperm cells after two rounds of division. In contrast, female meiosis is asymmetrical, meaning that only one oocyte will be produced, and the remaining three cells will be discarded as polar bodies. It has been postulated that centromeres can act as selfish genetic elements in female meiosis and favor their own transmission to the oocyte [36]. Homologous chromosomes with “stronger” centromeres would therefore be more likely to be passed to the progeny. This hypothesis is known as centromere drive, and is supported by observations of female-specific, non-Mendelian segregation of genetic markers in some species. An especially drastic example is found in the plant genus *Mimulus*. In heterozygous hybrids generated by crossing *M. guttatus* and *M. nasutus*, allele D (driver), contributed by *M. guttatus*, exhibits a nearly perfect transmission (98%) to the oocyte over its *M. nasutus* homolog [37]. Allele D was later shown to be linked to an unusually large centromeric region [38] supporting the hypothesis that chromosomes with larger centromeric regions are preferentially transmitted to the oocyte.

Mechanistic evidence for this phenomenon recently came from a study in mouse oocytes, where the authors showed how the number of minor satellite repeats at the centromere affects the likelihood of its transmission to the embryo [39,40]. When centromeres containing more repeats face the cortical (polar body) side, they are more likely to detach from microtubules and re-orient to face the oocyte side in anaphase I, thus favoring their segregation into the oocyte. This phenomenon is caused by the asymmetry of tyrosination on spindle microtubules [41].

Notably, strong female meiotic drive can cause fitness costs e.g. reduced male fertility [38]. The need to counteract these deleterious effects results in an evolutionary pressure on centromeric proteins to suppress centromere drive. The centromere drive hypothesis is therefore consistent with the observation that kinetochore and centromere proteins (including the centromeric histone variant, CENP-A) evolve faster than expected from their essential and conserved function [36,42]. Evolutionary studies provided evidence that CENP-A is under positive selective pressure in *M. guttatus, D. melanogaster*, and *A. thaliana* [43,44]. Interestingly, in species with symmetric female meiosis or holocentric centromeres no positive selective pressure on CENP-A was observed [45,46]. This was, for example, shown in several holocentric species in the plant *Luzula* genus.

In addition to centromere drive, centromere positioning may contribute to the evolution of centromere function in meiosis. Changes in centromere positioning were suggested to contribute to reproductive barrier establishment and speciation [47]. In a recent study, centromere repositioning induced by mutations in kinetochore proteins in fission yeast was shown to compromise meiotic divisions [48]. The authors observed an inverted order of segregation events in some cells homozygous for the repositioned centromere (separation of sister chromatids first), and an incompatibility between wild-type chromosomes and chromosomes with repositioned centromeres.

Taken together, the meiosis-specific phenomena, such as centromere drive, shape the function and potentially influence the structure of centromeric proteins. Since these proteins have essential roles in both mitosis and meiosis, it will be important to understand their specific adaptations to both processes.

## CENP-A adaptations for meiosis

**Figure 2:**
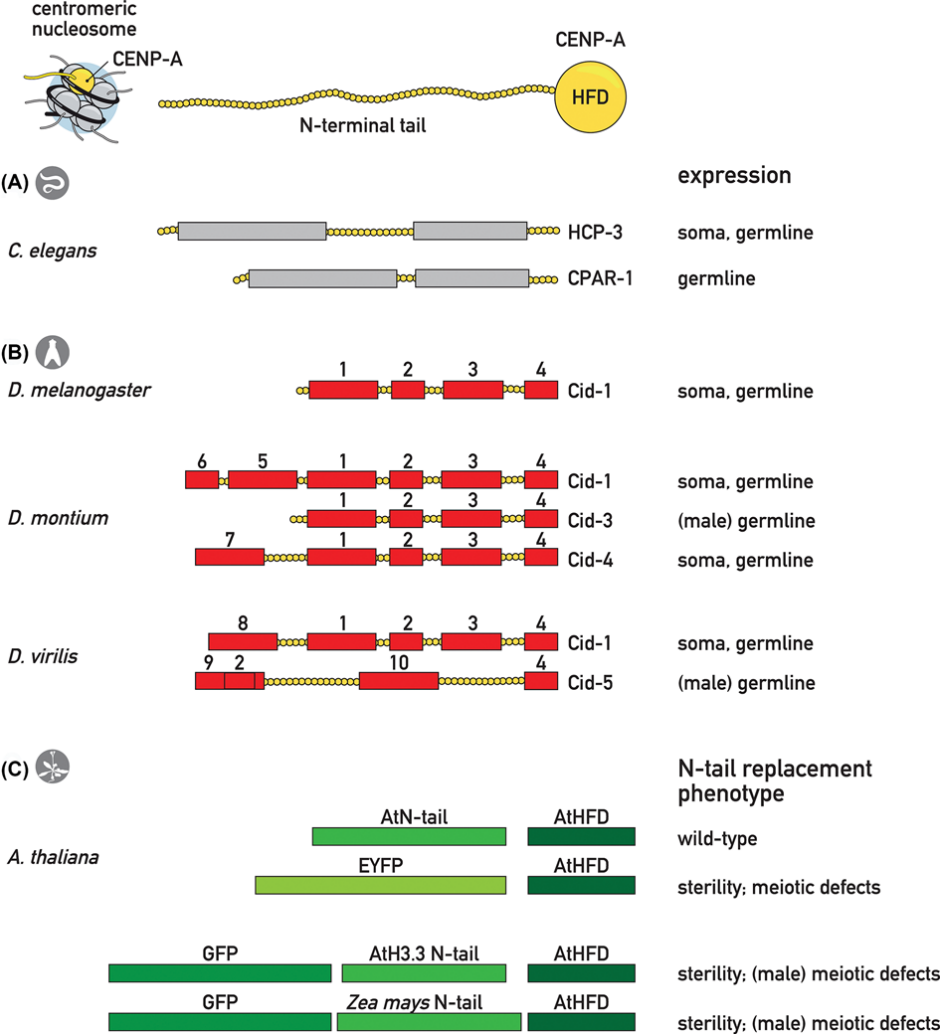
Rapid evolution of the CENP-A N-terminal tail may be linked to roles in the germ line (**A**) *Caenorhabditis elegans* contains two CENP-A paralogs (HCP-3 and CPAR-1), showing differential expression patterns. Only HCP-3 is essential for viability. (**B**) *Drosophila* lineages contain different CENP-A paralogs (called Cid) with variable tail sequences and differential expression patterns. The evolution of the tail sequences involved reshuffling of modules, as specific sequence motifs can be found in multiple lineages (numbered red boxes). (**C**) The N-terminal tail of *Arabidopsis thaliana* CENP-A/CENH3 is required for meiosis, and its replacement with EYFP, the AtH3.3 N-terminal tail, or the *Zea mays* CENH3 N-terminal tail results in sterility and meiotic defects. (**B**) adapted from [51]; (**C**) adapted from [58]; HFD, histone-fold domain.

CENP-A plays a key role for the segregation of chromosomes in both mitosis and meiosis in most organisms, but differences in the two processes may have shaped the evolution of its sequence and structure. CENP-A is composed of two regions: the histone-fold domain (HFD) and N-terminal tail. While the histone-fold domain, which incorporates into the core nucleosome, is very well conserved, the N-terminal tail is surprisingly divergent among species [49]. This is also true for closely related species, suggesting that this part of the protein is rapidly evolving (Figure 2). This evolution on the N-terminal tail is likely modular, since conserved sequence motifs were described both in *Drosophila* species as well as in a wide variety of plant species (Figure 2B,C) [50,51]. Strong evolutionary pressure on CENP-A might also explain the gene duplications in different species, often accompanied by the germ line-specific expression of one of the genes (Figure 2A,B). Gene duplicates are thought to allow fast evolution of factors with essential functions, whose mutations would otherwise be selected against. After the duplication, one of the genes can assume more specialized functions, whereas the other one still performs its initial role. Interestingly, CENP-A duplicates differ mostly in the N-terminal tail sequence [51–56].

Several studies have investigated the functional differences between duplicated CENP-A genes within the same species. In *Caenorhabditis elegans*, there are two genes encoding CENP-A paralogs: *hcp-3* and *cpar-1* (Figure 2A). They have different expression patterns, with CPAR-1 present mostly in the germline, and HCP-3 in all dividing cells in both the germline and embryos (Figure 2A). The sequences of both proteins differ mainly in the N-terminal part, where CPAR-1 is recognized by separase, resulting in the removal of most of the N-terminal tail at the metaphase–anaphase transition of meiosis I [53]. Interestingly, only HCP-3 is an essential protein and seems to be the *bona fide* CENP-A. However, while it is present on chromatin during both meiotic cell divisions, it is dispensable for meiosis, and the two meiotic divisions appear normal upon depletion of HCP-3 [52]. The function of CPAR-1 and the significance of the partial N-terminal tail cleavage during meiosis remain unknown.

Across the *Drosophila* genus, five paralogs of CENP-A were initially identified (called Cid1-5) [51]. Species from the *Sophophora* subgenus typically express three of them (Cid1, Cid3, and Cid4), whereas the *Drosophila* subgenus expresses two paralogs (Cid1 and Cid5). More recently, a novel Cid duplication was described for the *Drosophila* subgenus (Cid6) [56]. All the variants tested in *D. auraria* show centromeric localization, suggesting that all retained centromeric function [51]. Interestingly, in two species tested, the authors identified CENP-A paralogs restricted to the male germline: Cid3 in *D. auraria* and Cid5 in *D. virilis* (Figure 2B) [51]. Moreover, *D. virilis* Cid1 and Cid5 show differential expression profiles, with Cid1 present during female meiosis and Cid5 retained in sperm [55]. This specificity has been attributed to rapidly evolving domains within the N-terminal tails of the CENP-A paralogs (Figure 2B) [51]. Since the centromeric histones face different functional requirements and hence different selective pressure in female and male meiosis, the authors hypothesized that a system with a CENP-A paralog dedicated to each function allows an adaptation to these constraints [51,55]*.*

A recent study revealed that most mosquito species encode two CENP-A paralogs: mosqCid1 and mosqCid2 [54]. A third paralog (mosqCid3) is present only in species from the *Aedes* genus. Consistent with studies in *Drosophila*, the sequences of mosquito CENP-A proteins have diverged mostly in the N-terminal tail. The uniformly expressed mosqCid1 seems to evolve under positive selective pressure, suggesting a role as a centromere drive suppressor [54]. In addition, mosqCid2 is germline-specific and abundantly present in both female and male germline [54].

Divergent CENP-A N-terminal tail sequences can thus be associated with germline expression, but a functional role for the N-terminal tail within meiosis has so far only been described in *A. thaliana.* Lermontova and colleagues showed that when the N-terminal tail is replaced with an EYFP tag, the resulting fusion protein can support mitotic divisions, but causes sterility in plants due to abnormal meiotic divisions (lagging chromosomes in meiosis II and presence of micronuclei in pollen spores) (Figure 2C) [57]. These observations were confirmed by Ravi and colleagues, who showed that replacing the endogenous N-terminal tail of *A. thaliana* CENP-A with either a GFP-tagged H3.3 N-terminal tail or a *Zea mays* N-terminal tail caused meiocytes to go from prometaphase I straight to anaphase I, leading to meiotic errors [57,58]. In both cases, these defects probably resulted from insufficient retention of fluorescently tagged tailless variants on meiotic centromeres, as neither EYFP-CENP-A nor GFP-CENP-A was detectable on chromatin during meiotic divisions [58]. Although the presence of the EYFP/GFP tag may have influenced these results, the observations strongly suggest a role for the N-terminal tail in CENP-A loading specifically in meiosis.

The meiosis-specific role for the N-terminal tail of CENP-A in *A. thaliana* suggests that it interacts with loading factors that distinguish mitosis and meiosis. Such factors have yet to be described. Additional support for the existence of such loading factors comes from the observation that in some species, like rye and *D. melanogaster*, CENP-A loading in meiosis occurs at different times during the cell cycle compared to mitosis [59–61]. However, in *D. melanogaster*, it was shown that the factors required for CENP-A loading —CAL1 and CENP-C —are also important for its meiotic loading, despite the differences in the timing of incorporation [59,62]. Nevertheless, as the focus of centromere analysis shifts from the analysis of culture cells to the study of a more developmental context in model organisms, it is possible that new CENP-A chaperones with meiosis-specific roles will be uncovered in the future.

## CENP-A inheritance through meiosis

**Figure 3:**
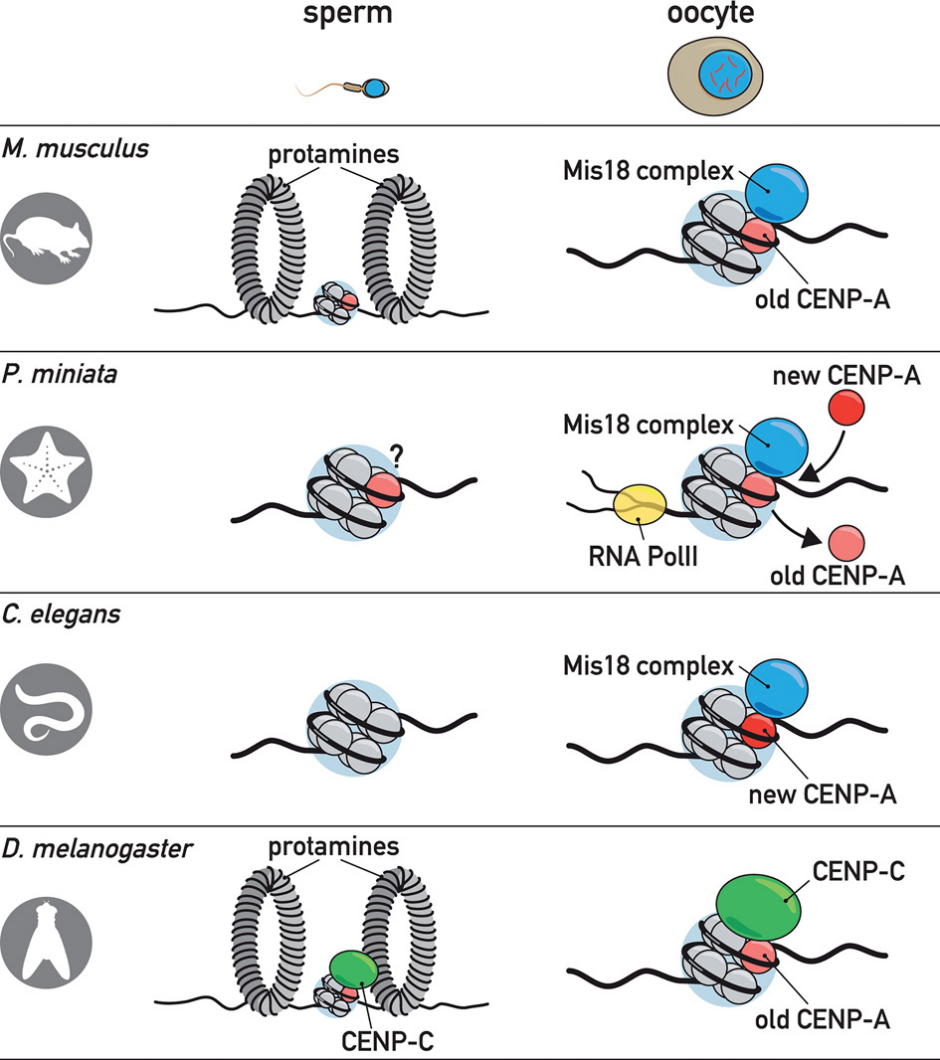
Diversity in the mechanisms of centromere inheritance and maintenance through the germ line CENP-A can be inherited through the paternal or maternal germ line, or both. In sperm, nucleosomes can be replaced by protamines that are depicted as large toroids. Molecules of CENP-A are either retained (*M. musculus*), constantly replaced at the same locus (*P. miniata*) or re-established during meiosis (*C. elegans*). Different protein factors associate with meiotic CENP-A, such as the MIS18 complex, KNL-2 (kinetochore null 2, a homolog of Mis18BP1), or CENP-C.

Recently, strides have also been made in understanding how the centromere, and more specifically CENP-A, is inherited from one generation to the next. Epigenetic information in the form of histone modifications or histone variants are frequently transmitted through both maternal or paternal gametes [63,64]. Interestingly, CENP-A presence in gametes differs markedly across species. It can be present in both mature oocytes and sperm (e.g*. D. melanogaster*), solely in mature oocytes (e.g. *C. elegans*), or only in mature sperm (e.g. *A. thaliana*) (Figure 3) [52,60,65,66]. For *D. melanogaster*, it was shown that altered CENP-A levels in mature sperm affect the levels of CENP-A in fertilized embryos. In addition, removing CENP-A from mature sperm led to a failure of embryonic CENP-A loading on paternal chromosomes, arguing that CENP-A presence in mature sperm is required for inheritance of centromere identity [60]. For species that lack CENP-A in either one of the germ cells, centromeres have to be established *de novo* in the embryo. However, mechanistic insight into this process is currently lacking.

If the presence of CENP-A in germ cells is important for passing on centromere identity, it needs to be maintained in the context of meiosis, even if it is not always required for meiotic chromosome segregation. How CENP-A is inherited through the germ line differs between lineages. In mouse oocytes, CENP-A is retained at the centromeric sites, and the protein is stable enough to maintain centromere identity and function for the entire lifespan of the female mouse without any detectable turnover of the protein (Figure 3) [67]. This may not be the case in all species, however, as illustrated by a recent study in starfish *(Patiria miniata)* oocytes. In this model organism, oocytes can be maintained in culture for long periods of time, during which CENP-A is continuously recycled at the centromere (Figure 3) [68]. This process was shown to be dependent on active transcription (RNA Pol II) and loading factors that are also involved in CENP-A maintenance during mitosis (Mis18 complex). Some species, like *C. elegans*, lose CENP-A during early meiosis and completely re-establish it later on in prophase I (Figure 3) [52,65]. The functional significance of the removal and re-incorporation of CENP-A in *C. elegans* has yet to be determined. Nonetheless, it clearly demonstrates that several different solutions have arisen during the course of evolution to ensure the inheritance of centromere identity in future generations: CENP-A is either retained, recycled or re-established in meiosis.

## Outlook

The inherent challenge of studying meiosis is the necessity to describe it within the context of a whole organism. Despite this important limitation, the differences in meiotic centromere organization, behavior, and dynamics have been described in recent years, in a variety of model organisms, revealing that centromeres have undergone a range of adaptations to fulfill meiosis-specific functions. Meiotic centromeres act as important genomic elements that help orchestrate meiotic events, such as pairing and recombination. It will be interesting to further explore the meiotic roles of the centromere that go beyond chromosome segregation.

A lot of evidence has recently been produced in favor of the centromere drive hypothesis that views the centromere as a selfish genetic element. More mechanistic studies will reveal how this centromere feature shaped the regulation of meiotic chromosome segregation, and how centromeres have responded to this constraint. These changes probably differ substantially between and even within different lineages. Evolutionary pressure to adapt centromeres appears to have shaped the N-terminal tail of CENP-A in particular. It remains unclear, however, whether changes in this protein domain have led to novel interacting partners and therefore novel functions. One such function could be to ensure the maintenance of centromere identity by affecting the inheritance of CENP-A through meiosis. For now, different model organisms have been described in which CENP-A is either retained, recycled or re-established. Future studies on meiotic centromeres should aim to uncover the general principles behind these adaptive changes.

## Summary

- The meiotic centromere plays a crucial role in coordinating homologous recombination and in the stepwise segregation of chromosomes to produce haploid gametes, through mechanisms that are absent in mitosis.
- Mechanistic insights into how meiotic centromeres act as selfish genetic elements may explain differences in centromeric organization between species.
- The N-terminal tail of CENP-A, the histone variant that marks centromeres, rapidly evolves, and changes in its sequence probably support protein–protein interactions with meiosis-specific factors.
- CENP-A is retained, recycled, or re-established in meiosis in different lineages to define centromere identity for the following generation.

## Abbreviations

DSB: double stranded break
HFD: histone-fold domain
